# Supplementary material for: Hybrid deep reconstruction for vignetting-free upconversion imaging through scattering in epsilon-near-zero materials
Source: Light Sci Appl. 2026 Jul 21;15:327. doi: 10.1038/s41377-026-02375-6 (PMC13389204; doi:10.1038/s41377-026-02375-6)
Supplement: Supplementary file 1 — SUPPLEMENTAL INFORMATION [file 41377_2026_2375_MOESM1_ESM.docx]

**Hybrid Deep Reconstruction for Vignetting-Free Upconversion Imaging through Scattering in Epsilon-near-zero Materials**

Hao Zhang^*1, 2,†^, Yang Xu^3, †^, Wenwen Zhang^4^, Saumya Choudhary^5^, M. Zahirul Alam^6^, Long D. Nguyen^3^, Matthew Klein*^7^*, Shivashankar Vangala*^7^*, J. Keith Miller*^8^*, Eric G. Johnson*^9^*, Joshua R. Hendrickson*^7^*, Robert W. Boyd^3,5,6^ and Sergio Carbajo^1, 2, 10, 11^

*^1^Department of Electrical and Computer Engineering, UCLA, 420 Westwood, Los Angeles, CA 90095, USA*

*^2^SLAC National Accelerator Laboratory, Stanford, 2575 Sand Hill Rd, Menlo Park, CA 94025, USA*

*^3^Department of Physics and Astronomy, University of Rochester, 500 Wilson Blvd, Rochester, New York 14627, USA*

*^4^David Geffen School of Medicine, UCLA, 10833 Le Conte Ave, Los Angeles, CA 90095, USA*

*^5^The Institute of Optics, University of Rochester, 480 Intercampus Dr, Rochester, New York 14627, USA*

*^6^Department of Physics, University of Ottawa, 150 Louis-Pasteur Private, Ottawa, Ontario K1N 6N5, Canada*

*^7^Sensors Directorate, Air Force Research Laboratory, Wright-Patterson AFB, Dayton, OH 45433, USA*

*^8^The Holcombe Department of Electrical and Computer Engineering, Clemson Center for Optical Materials Science and Engineering Technologies, 300 S Palmetto Blvd, Clemson, SC 29634, USA*

*^9^CREOL, The College of Optics and Photonics at the University of Central Florida, 4304 Scorpius St, Orlando, FL 32816, USA*

*^10^Physics and Astronomy Department, UCLA, 475 Portola Plaza, Los Angeles, CA 90095, USA*

*^11^California NanoSystems Institute, 570 Westwood Plaza, Los Angeles, CA 90095, USA*

Corresponding author: *[haozh@g.ucla.edu](mailto:haozh@g.ucla.edu); ^†^equal contribution

**Supplementary Note 1: Epsilon-near-zero (ENZ) sample**

The sample used in the experiment consists of a 310-nm-thick ITO film deposited on a sapphire substrate. The ITO layer was fabricated by RF sputtering, and its measured resistivity is approximately 1.7 × 10⁻⁴ Ω·cm.

Field enhancement: The electric field enhancement inside the indium tin oxide (ITO) layer was numerically calculated based on the Drude model. The dielectric function of ITO can be expressed as:

$$\varepsilon(\omega)=\varepsilon_{\infty}-\frac{\omega_{p}^{2}}{\omega^{2}+i\omega\gamma}$$

where $\varepsilon_{\infty}$ is the high-frequency permittivity, $\omega_{p}$ is the plasma frequency, and $\gamma$ is the damping constant. Under this condition, the permittivity approaches zero at the ENZ wavelength (λ = 1510 nm), leading to strong localization of the electric field inside the ITO layer. Numerical simulations were performed using the transfer-matrix method.

Figure X shows the simulated electrical field intensity enhancement as a function of the incident angle at λ = 1510 nm. Under the ideal ENZ condition, the calculated field intensity enhancement reaches approximately **90×**, confirming the strong field confinement effect characteristic of ENZ materials.


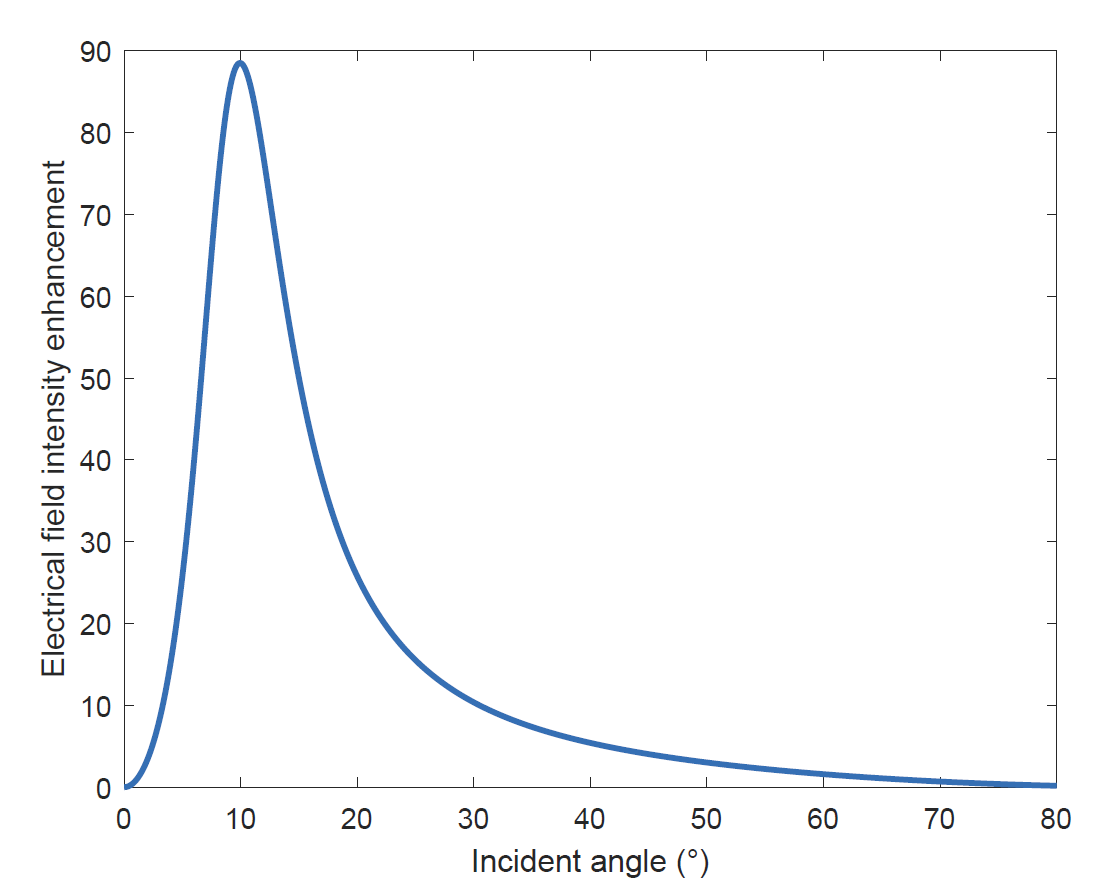


Figure S1. Field enhancement near ENZ wavelength we used in the experimental setup.

**Supplementary Note 2: Experimental Configurations**

**Gate pulse characteristics**

Both the pump and probe pulses have a duration of 100 fs. The gating beam is incident on the ITO layer at an angle of 12° relative to the image-carrying signal beam. Without additional pulse-front-tilt correction, the effective width of the nonlinear interaction window is measured to be approximately 60 µm. The absolute conversion efficiency of the FWM process exhibits a quadratic dependence on the gating-pulse power. In our current experimental setup, the measured conversion efficiency is 1.25 × 10⁻⁵.

**Scattering Conditions**

For the USAF target experiments, three concentrations of polystyrene microsphere suspensions (0.175, 0.35, and 0.525 g/cm³) were applied to simulate amplitude scattering. Each suspension was manually agitated prior to data acquisition to redistribute the microspheres, ensuring a randomized scattering configuration for every measurement. Although each acquisition represents a static snapshot, the overall dataset captures multiple independent scattering realizations, mimicking a quasi-dynamic scattering environment.

The optical powers were adjusted across scattering levels to maintain detectable upconverted signals:

- Pump beam: 22 mW (constant)
- Probe beam: 8.6 mW (0.175 g cm^-3^), 16 mW (0.35 g cm^-3^), and 34 mW (0.525 g cm^-3^)

For the OAM target experiments, three optical diffuser configurations were used—600 grit, 1500 grit, and a combined dual-grit (1500 & 600)—to introduce controlled phase distortions and surface scattering. During acquisition, the diffusers were continuously rotated to emulate time-varying scattering dynamics, analogous to realistic motion-induced speckle variations encountered in biological or dynamic optical media.

The corresponding optical powers were:

- Pump beam: 13 mW (constant)
- Probe beam: 1.4 mW (1500 grit), 6.8 mW (600 grit), and 8 mW (dual 1500 & 600 grit)


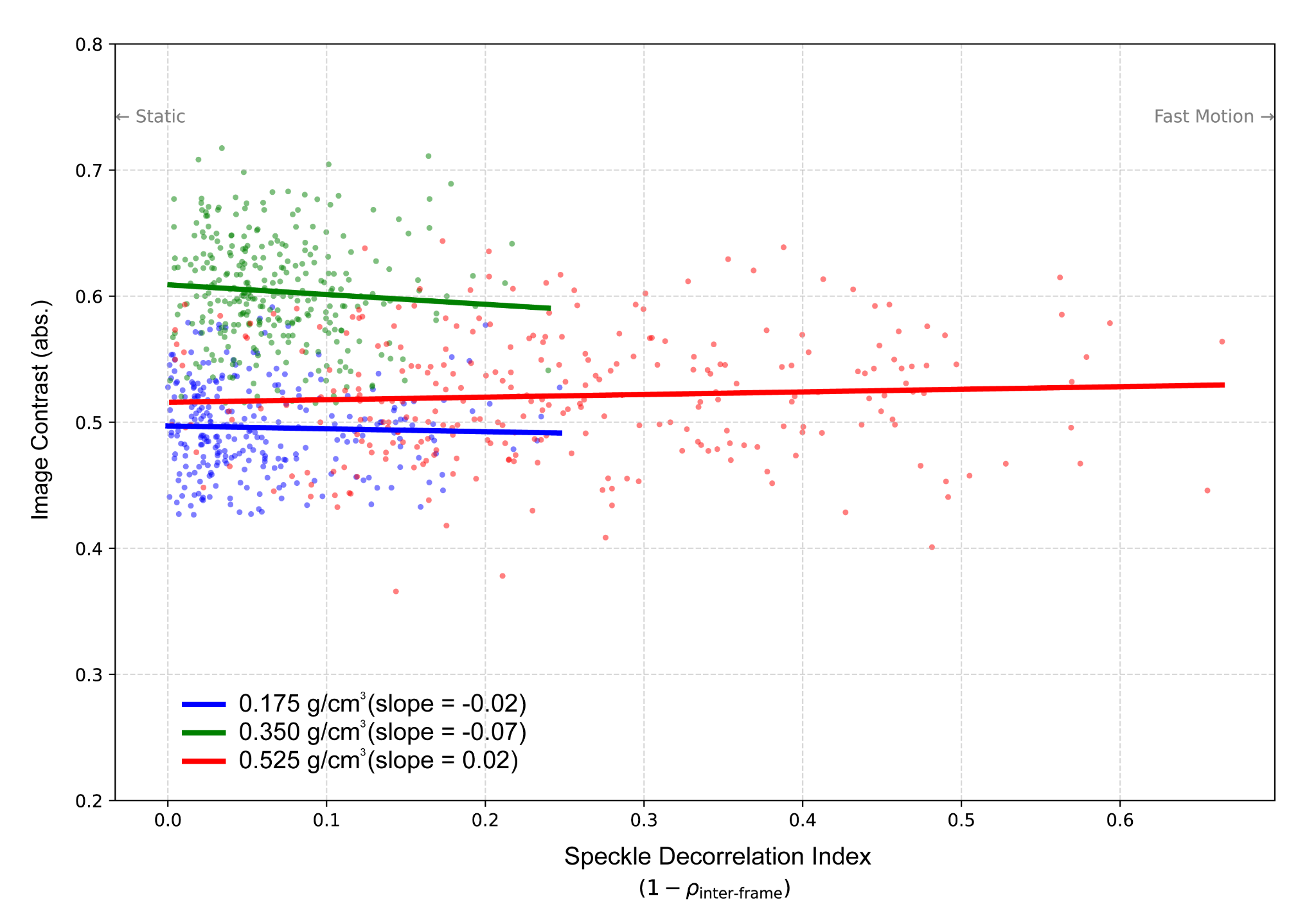


Figure S2: Evaluation of reconstruction robustness against particle motion dynamics. The scatter plot correlates the absolute image contrast with the particle motion intensity, quantified by the inter-frame speckle decorrelation index ($1-\rho$), for three scattering densities: 0.175 g cm^-3^ (blue), 0.350 g cm^-3^ (green), and 0.525 g cm^-3^ (red). The data points represent individual video frames recorded during the settling process, capturing a transition from high motility (right) to a quasi-static state (left).

We adopted the inter-frame Speckle Decorrelation Index (1 - \rho) as a robust, quantitative proxy for particle motion intensity, consistent with standard dynamic light scattering principles. We analyzed the correlation between the reconstruction contrast and the particle motion intensity across three different scattering concentrations: 0.175 g cm^-3^, 0.350 g cm^-3^, and 0.525 g cm^-3^. As illustrated in Figure S2, our data covers a wide dynamic range, with the motion index extending up to 0.65 in the strongest scattering regime, indicating extremely rapid dynamic scattering changes. Crucially, linear regression analysis performed on these scatter plots reveals consistently near-zero slopes across all three regimes (-0.02, -0.07, and +0.02, respectively).

**Supplementary Note 3: Evaluation Matrics**

We performed a comprehensive evaluation of the reconstruction performance under three different scattering conditions on two input patterns, using the peak signal-to-noise ratio (PSNR), structural similarity index (SSIM), and intersection over union (IoU) as quantitative metrics.

**PSNR** is a measure of the reconstruction fidelity, quantifying the ratio between the maximum possible value and the power of corrupting noise, which can be defined as:

$PSNR = 10\cdot{Log}_{10}({Max}_{I}/MSE)$ (1)

where ${Max}_{I}$ is the maximum possible pixel value and MSE is the mean squared error between the predicted and reference images. Higher PSNR shows better reconstruction performance.

**SSIM** assesses perceived image quality by comparing structural information, luminance and contrast between the reconstructed and refereced images, which can be defined as:

$SSIM(x,y) =\frac{[(2\mu_{x}\mu_{y}+C_{1})(2\sigma_{xy}+C_{2})]}{(\mu_{x}^{2}+\mu_{y}^{2}+C_{1})(\sigma_{x}^{2}+\sigma_{y}^{2}+C_{2})}$ (2)

where $\mu_{x}$ and $\mu_{y}$ are the local means of the reconstructed images and reference images, $\sigma_{x}^{2}$ and $\sigma_{y}^{2}$ are the local variances and $\sigma_{xy}$ is the local covariance. $C_{1}$ and $C_{2}$ are constants, which are used to stabilize the division with weak denominators. SSIM typically ranges from 0 to 1, where 1 indicates perfect structural similarity and values closer to 0 suggest low similarity.

**IoU** quantifies the overlap between the predicted and true binary masks. It is computed as:

$IoU = TP/(TP+FP+FN)$(3)

where TP, FP, and FN represent the numbers of true positives, false positives, and false negatives, respectively. Higher IoU reflects better spatial correspondence.

**Effective field-of-view (eFOV):** The effective field-of-view (eFOV) is defined as the image region that retains meaningful structural information after reconstruction, rather than the physical or optical coverage of the imaging system. To quantify the relative expansion of this effective FOV, both the raw FWM-scattering and DeepTimeGate-reconstructed images were analyzed. Each image was first converted to grayscale and segmented using Otsu’s adaptive thresholding (with a relaxed threshold factor of 0.65) to isolate regions containing recoverable structural signal. The resulting binary mask was refined by connected-component analysis, and the largest contiguous bright region was enclosed by its minimum bounding rectangle. The area of this rectangle, denoted as A, was defined as the effective FOV area for that image. DeepTimeGate expanded the effective FOV by approximately 45% relative to the raw FWM-scattering image (shown in Figure S3). The relative improvement in eFOV provided by DeepTimeGate compared with the baseline FWM-scattering image was computed as:

$$Effective FOV improvement = \frac{A_{DeepTimeGate} -A_{FWM}}{A_{FWM}}$$

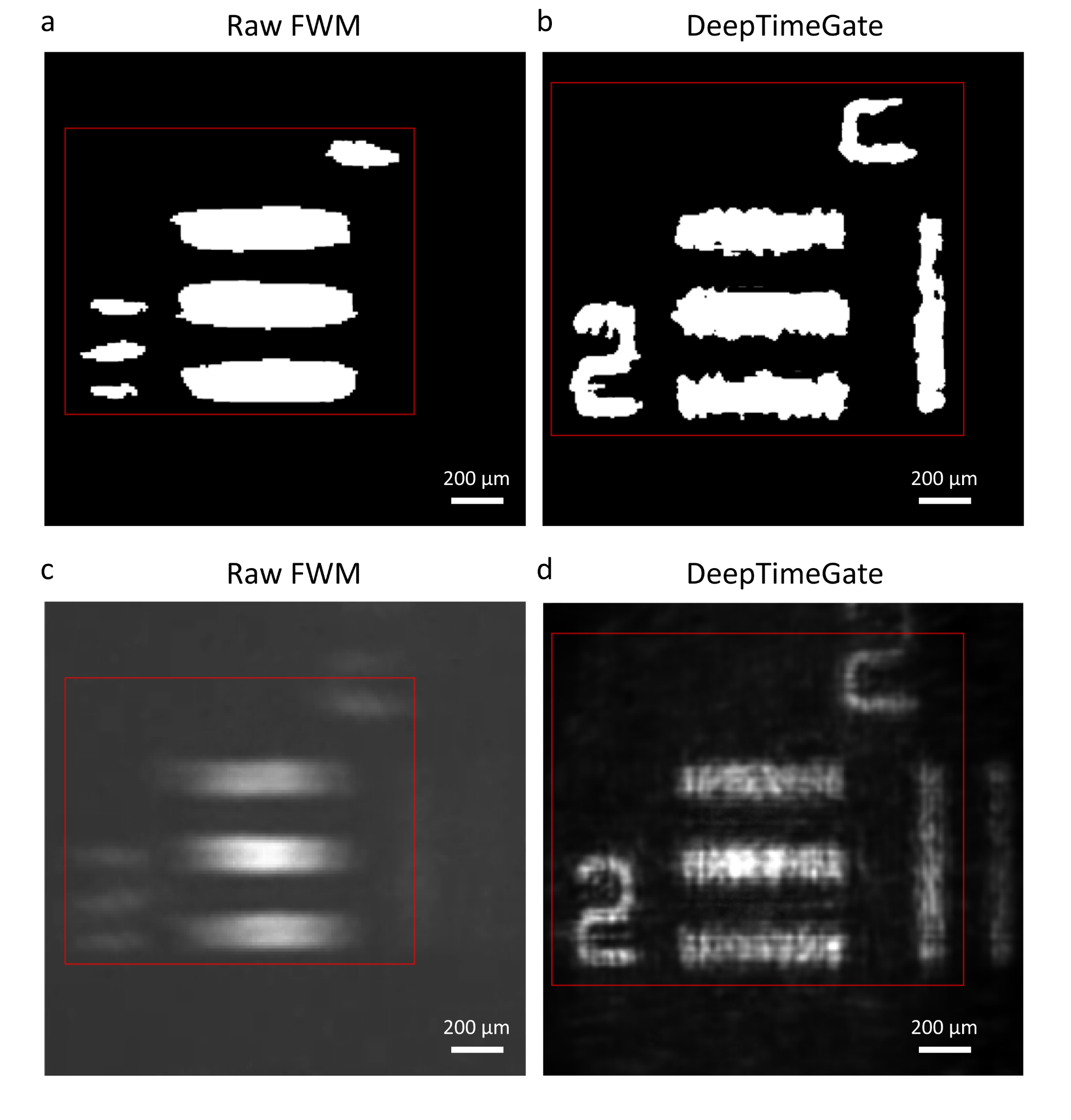


Figure S3. Comparison of binary and greyscale reconstructions. (a) Raw FWM binary output. (b) DeepTimeGate binary output. (c) Raw FWM greyscale image. (d) DeepTimeGate greyscale reconstruction. Red boxes mark the minimum bounding rectangles defining the effective FOV. DeepTimeGate expands the eFOV by ~45.5% compared with FWM.

**Spatial sharpness improvement:** To quantitatively evaluate the improvement in spatial sharpness, the intensity profiles along the horizontal and vertical midlines of the central bright bar were extracted from both the FWM-scattering and DeepTimeGate-reconstructed images. Edge sharpness was quantified using the 10–90% intensity rise width, which measures the spatial distance between the 10% and 90% points of the normalized edge transition. Smaller values indicate sharper and less blurred edges. As shown in Figure S4, DeepTimeGate reconstruction exhibited a 58% reduction in average horizontal edge width and a 3% reduction in vertical edge width relative to the raw FWM-scattering image. To demonstrate the method's performance on a spatially symmetric target, we have included the OAM results in the Figure S5.


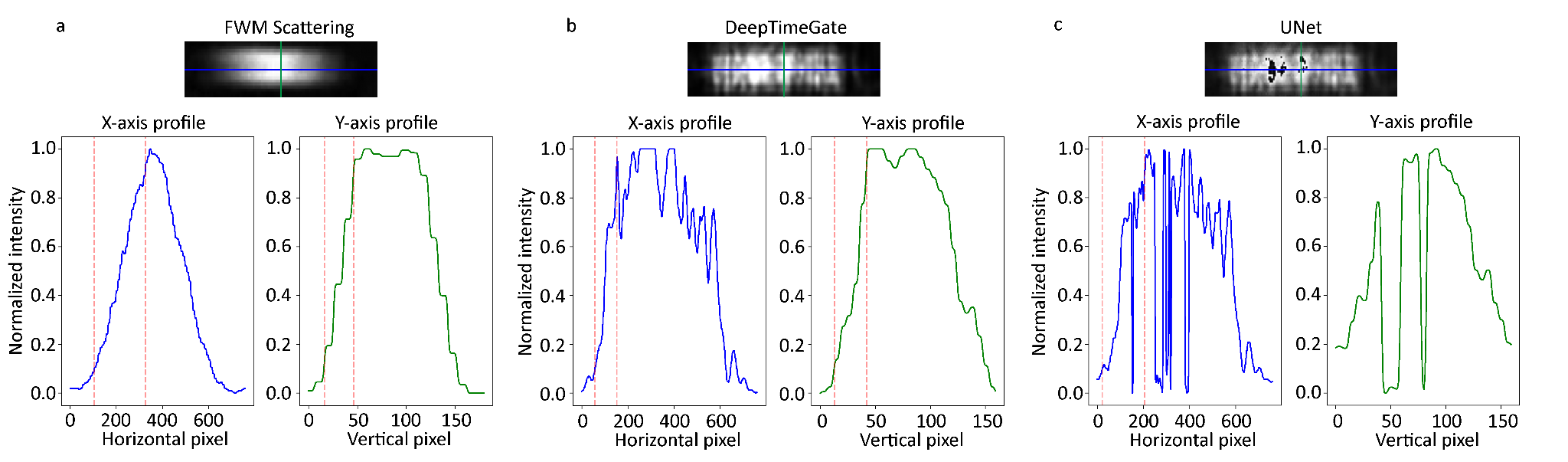


Figure S4. USAF image spatial sharpness analysis of FWM scattering (a) and DeepTimeGate (b) images. Intensity profiles along horizontal (blue) and vertical (green) midlines show the 10–90% rise width (dashed lines) used to quantify edge sharpness.


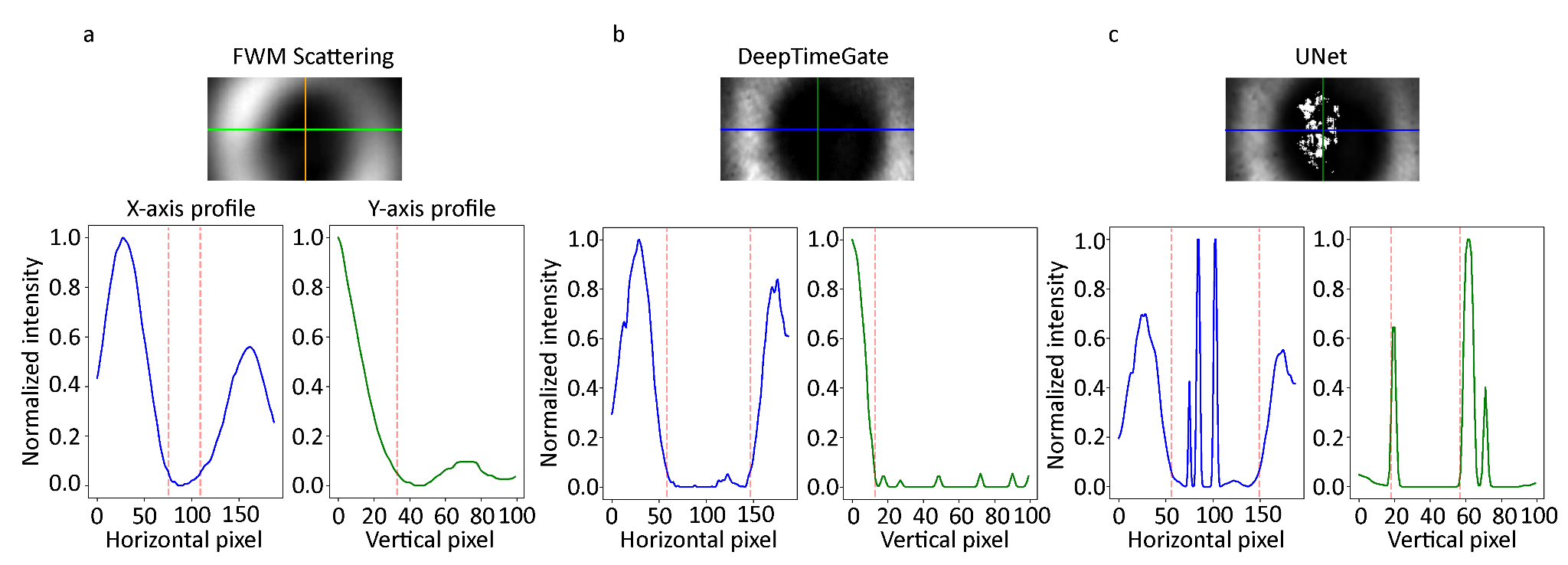


Figure S5. OAM image spatial sharpness analysis of FWM scattering (a) and DeepTimeGate (b) images. Intensity profiles along horizontal (blue) and vertical (green) midlines show the 10–90% rise width (dashed lines) used to quantify edge sharpness.

**Supplementary Note 4: Defect analysis**

As presented in the Figure S6, we employed box plot analysis to visualize the statistical dispersion of pixel intensities, which serves as a robust proxy for image continuity and defect density. In this statistical context, "structural continuity" is quantified by the tightness of the intensity distribution, while "defect spots", such as dead pixels or fragmented holes within a bright structure, manifest as statistical outliers (low-intensity values) that deviate significantly from the structural mean.

Referring to the comparative analysis in Figure S6, the U-Net reconstruction exhibits a severely fragmented intensity distribution, characterized by a heavy "long tail" of low-intensity pixels. Specifically, our analysis identifies 2,042 statistical outliers (accounting for 8.51% of the ROI) and a significant signal void ratio of 12.91%, which corresponds directly to the visually apparent "dead pixels" or broken structures. In stark contrast, the DeepTimeGate reconstruction effectively eliminates these artifacts, reducing the number of statistical outliers to zero and dropping the void ratio to 4.01%. The "smoothness" and background suppression capabilities are strikingly evident in the statistical distribution of the OAM target (Fig. S6d-f). The U-Net reconstruction exhibits a highly dispersed, long-tailed distribution, as indicated by a significant divergence between its mean (31.21) and median (3.00) pixel intensities. This widespread indicates substantial background noise and inconsistent signal levels. Conversely, the DeepTimeGate reconstruction achieves a highly compact and converged distribution (Mean: 5.34, Median: 1.00) with a tighter Interquartile Range (IQR = 8.00 vs U-Net's 11.00). This drastic reduction in statistical dispersion proves that our method effectively suppresses background artifacts and yields a structurally smoother, more uniform reconstruction compared to the noisy baseline.


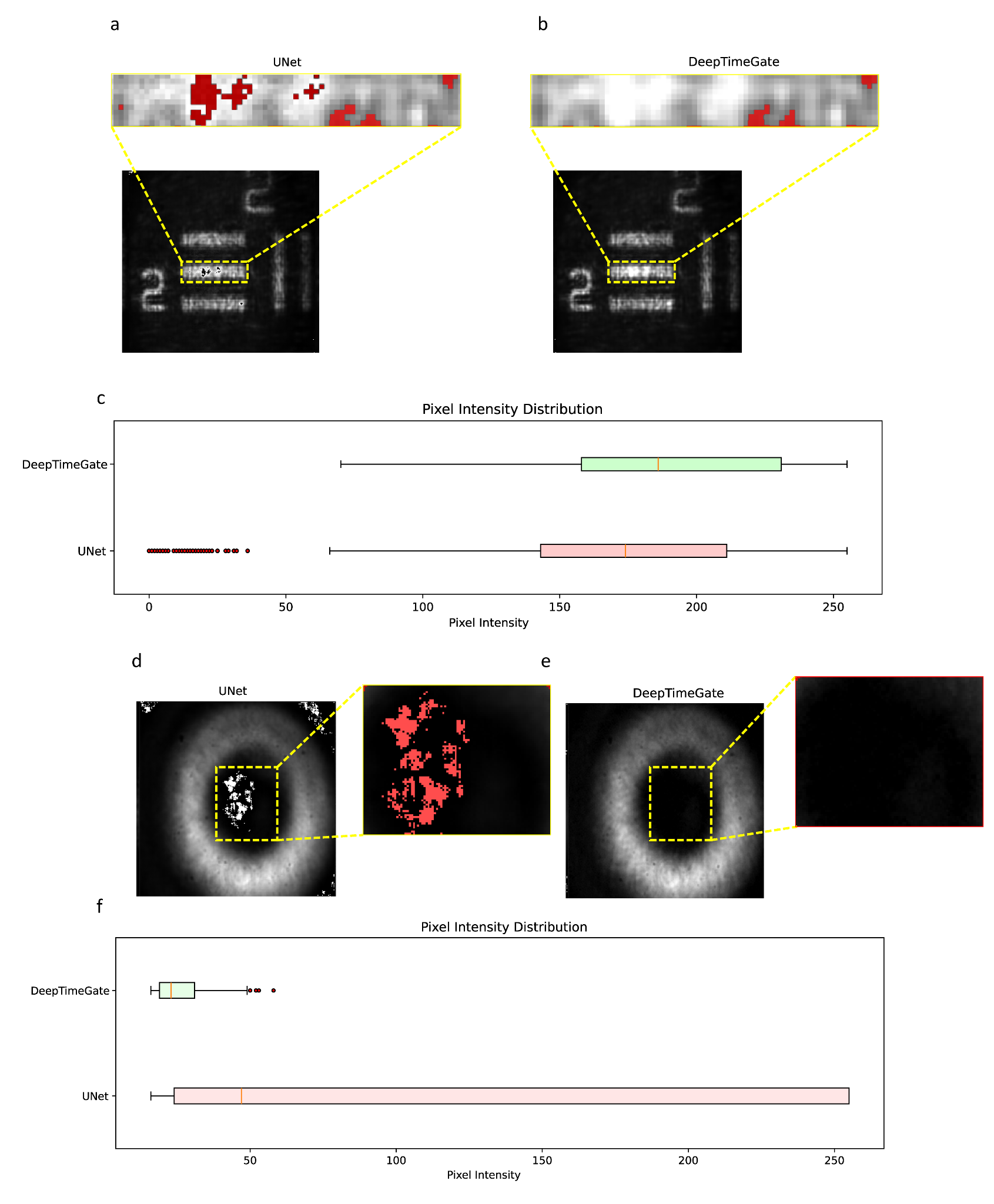


Fig S6. Analysis of defect reduction and structural continuity. (a-b) Comparison of reconstruction quality on the resolution target. Red overlays in (a) highlight "defect spots" present in the standard U-Net output, which are corrected in the DeepTimeGate result (b). (c) Pixel Intensity Distribution: statistical analysis of the highlighted ROI. The U-Net distribution (pink) shows a large spread (roughness) and numerous outliers near zero (defect spots). The DeepTimeGate distribution (green) is tighter (smoother) and free of low-intensity outliers. (d-e) Comparison on the OAM target. The U-Net output suffers from severe fragmentation (red highlights), while DeepTimeGate restores structural continuity. (f) Corresponding box plot showing the significant reduction in variance and elimination of outlier artifacts by the DeepTimeGate method.

**Supplementary Note 5: Examples of reconstruction comparison**

**
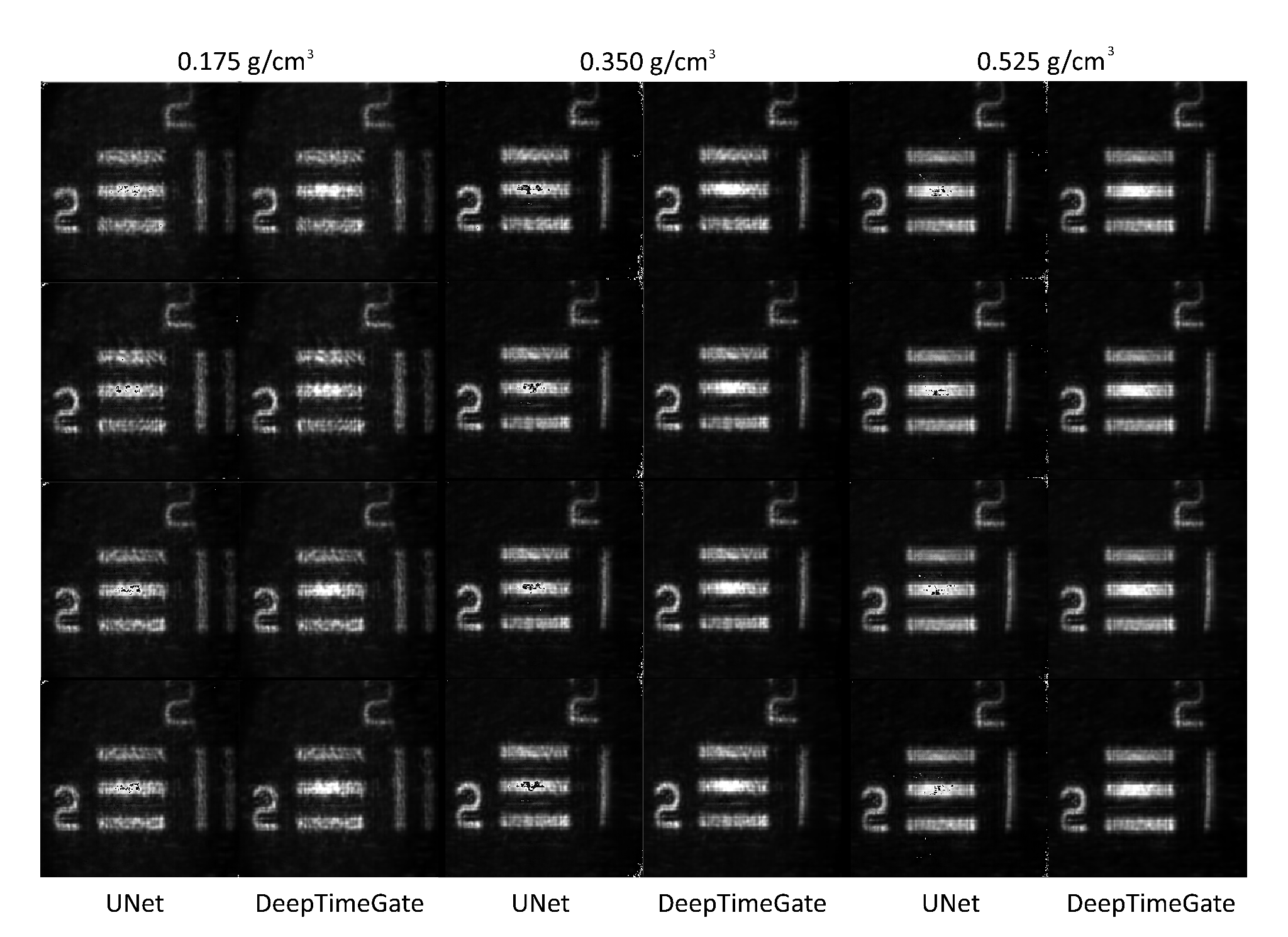
**Figure S7. Examples of UNet/DeepTimeGate reconstruction results from the USAF resolution chart under varying scattering strengths.**
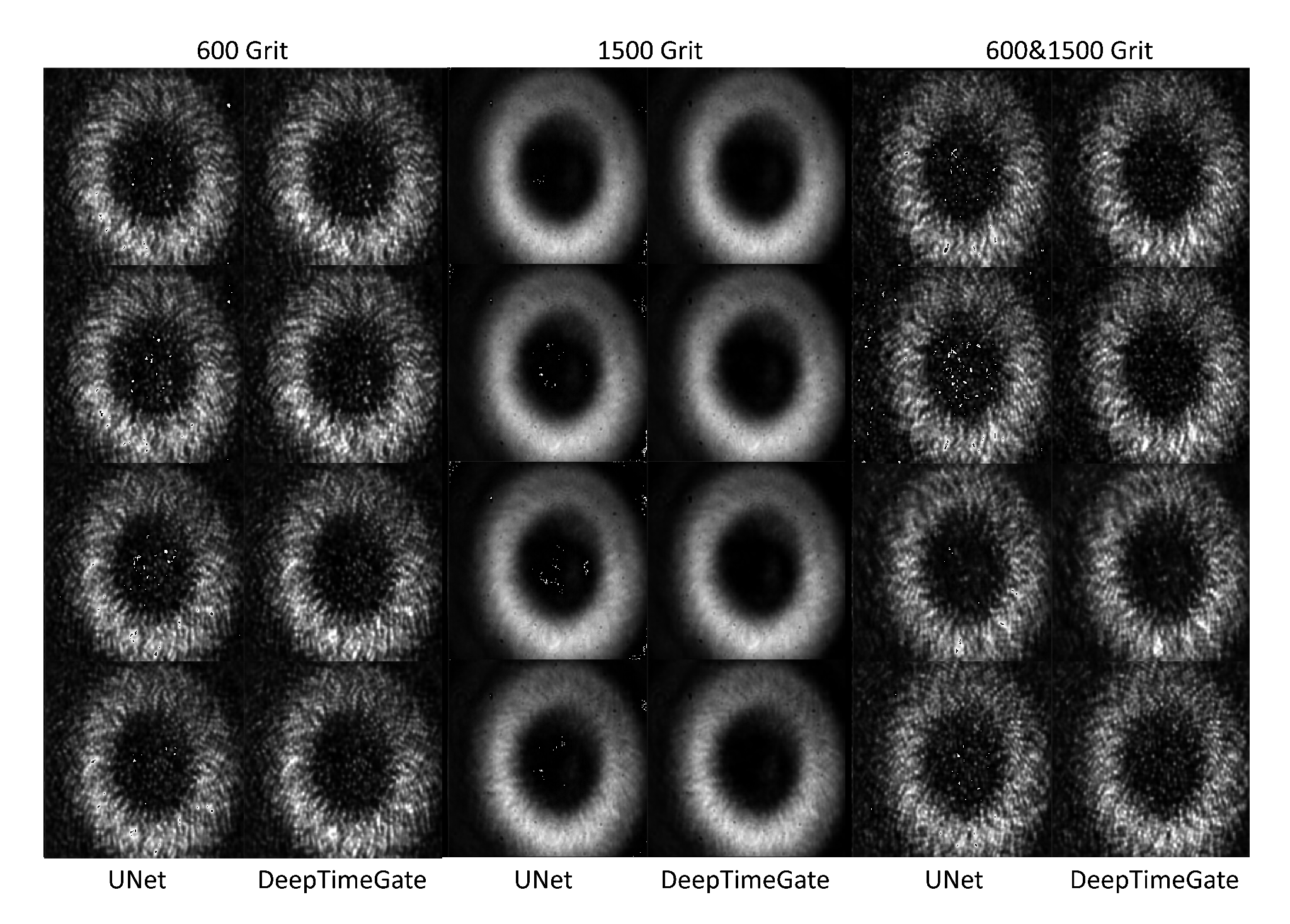
**

Figure S8. Examples of UNet/DeepTimeGate reconstruction results from the USAF resolution chart and vortex-phase OAM targets (right) under varying scattering strengths.
