# Supplementary material for: Hybrid deep reconstruction for vignetting-free upconversion imaging through scattering in epsilon-near-zero materials
Source: Light Sci Appl. 2026 Jul 21;15:327. doi: 10.1038/s41377-026-02375-6 (PMC13389204; doi:10.1038/s41377-026-02375-6)
Supplement: Supplementary file 3 — 41377_2026_2375_MOESM3_ESM [file 41377_2026_2375_MOESM3_ESM.docx]

**Video 1–3:**

- **USAF targets:** Three concentrations of polystyrene microsphere suspensions (0.175 g cm^-3^, 0.35 g cm^-3^ and 0.525 g cm^-3^) were applied to simulate increasing levels of volumetric scattering. Each video corresponds to one concentration, showing the imaging performance as scattering strength increases from low to high.

**Video 4–6:**

- **OAM targets:** Three diffuser configurations were used to introduce varying degrees of surface scattering: a single-layer 600-grit diffuser, a single-layer 1500-grit diffuser, and a dual-grit combination consisting of 1500-grit followed by 600-grit. Each video demonstrates how the OAM beams interact with these different diffuser setups.
